# Flexible Bronchoscopic Retrieval of an Aspirated Toy Whistle Using a Modified 4 Fr Fogarty Balloon Technique

**DOI:** 10.7759/cureus.110285

**Published:** 2026-06-05

**Authors:** Eldhos Jacob, Srishankar Bairy, Jason G Dsouza, Carishma S, Basil B Mathews

**Affiliations:** 1 Respiratory Medicine, Father Muller Medical College Hospital, Mangaluru, IND; 2 Paediatrics, Father Muller Medical College Hospital, Mangaluru, IND

**Keywords:** atelectasis, flexible bronchoscopy, fogarty catheter, foreign body aspiration, pediatric bronchoscopy

## Abstract

Foreign body aspiration in children is an important cause of airway morbidity and may present with persistent cough, delayed diagnosis, atelectasis, and granulation tissue formation. Smooth hollow plastic foreign bodies may be particularly difficult to retrieve because standard forceps often fail to achieve a secure grip, and balloon-assisted techniques may be limited by repeated slippage.

We report the case of a seven-year-old boy who presented with a persistent cough for 15 days following aspiration of a toy whistle. He was referred to our center after an unsuccessful retrieval attempt at another hospital in South India. Chest radiography at our institution demonstrated left lung volume loss with compensatory contralateral hyperinflation, while computed tomography performed at the referring center demonstrated an endobronchial foreign body in the left main bronchus with lingular atelectasis. Flexible bronchoscopy was selected as a staged minimally invasive approach with rigid bronchoscopy backup immediately available. The procedure was performed under general anesthesia through a laryngeal mask airway using a Fujifilm EB-710P slim bronchoscope (Fujifilm, Tokyo, Japan) with a 2.2 mm working channel. During the initial bronchoscopy session, multiple whistle fragments were removed using alligator and rat-tooth forceps; however, a retained hollow cylindrical component could not be extracted because granulation tissue and retained secretions limited visualization. Nebulized budesonide was administered between procedures as part of institutional clinical practice to reduce airway inflammation and improve visualization, and repeat bronchoscopy was performed the following day. During the second session, a 4 Fr Fogarty balloon catheter was advanced distal to the retained component. Balloon inflation with air repeatedly failed because of slippage within the smooth lumen, whereas inflation with 0.5 mL saline achieved improved anchorage and enabled successful extraction. Follow-up imaging demonstrated complete lung re-expansion.

This case highlights the importance of individualized procedural planning and a staged bronchoscopic approach in technically difficult pediatric airway foreign bodies. Saline inflation of a Fogarty balloon catheter may represent a practical troubleshooting modification in selected hollow airway foreign bodies when conventional retrieval techniques fail.

## Introduction

Foreign body aspiration (FBA) in children is a common and potentially serious clinical problem associated with considerable respiratory morbidity. Clinical presentation ranges from acute respiratory distress to subtle manifestations such as persistent cough, wheeze, or recurrent respiratory symptoms, and delayed diagnosis may result in complications including atelectasis, recurrent infection, airway inflammation, and granulation tissue formation [1-3].

Rigid bronchoscopy remains the standard approach for pediatric airway foreign body retrieval because it provides superior airway control, ventilation, and access to a wide range of extraction instruments. However, flexible bronchoscopy has an increasingly recognized adjunctive role in selected situations, particularly when distal airway visualization is required, retrieval is technically difficult, or a staged minimally invasive approach is feasible. Several studies have demonstrated the safety and effectiveness of flexible bronchoscopy when performed by experienced operators, often with rigid bronchoscopy available as backup [4-7].

Smooth, hollow plastic foreign bodies are especially challenging because standard forceps may fail to obtain a secure grip, while balloon-assisted retrieval techniques may be limited by repeated slippage within smooth luminal surfaces. Previously described approaches for hollow or perforated foreign bodies include forceps-based extraction, Fogarty balloon catheter-assisted retrieval, and “pass-through” techniques in which the catheter is advanced distal to the foreign body and inflated to facilitate traction [8]. However, procedural modifications may occasionally be necessary when conventional techniques fail in technically difficult cases.

We report a staged flexible bronchoscopic retrieval of an aspirated toy whistle in a child in whom conventional forceps and air-inflated Fogarty balloon techniques were unsuccessful. Successful extraction was ultimately achieved using a saline-inflated 4 Fr Fogarty balloon catheter as a practical troubleshooting modification in a technically challenging hollow airway foreign body.

## Case presentation

A seven-year-old boy presented with a persistent dry cough for 15 days following aspiration of a toy whistle. He was initially evaluated at another hospital in South India, where computed tomography (CT) of the chest was performed and an attempted bronchoscopic retrieval was unsuccessful. The child was subsequently referred to our center with the CT images and report for further management.

**Figure 1:**
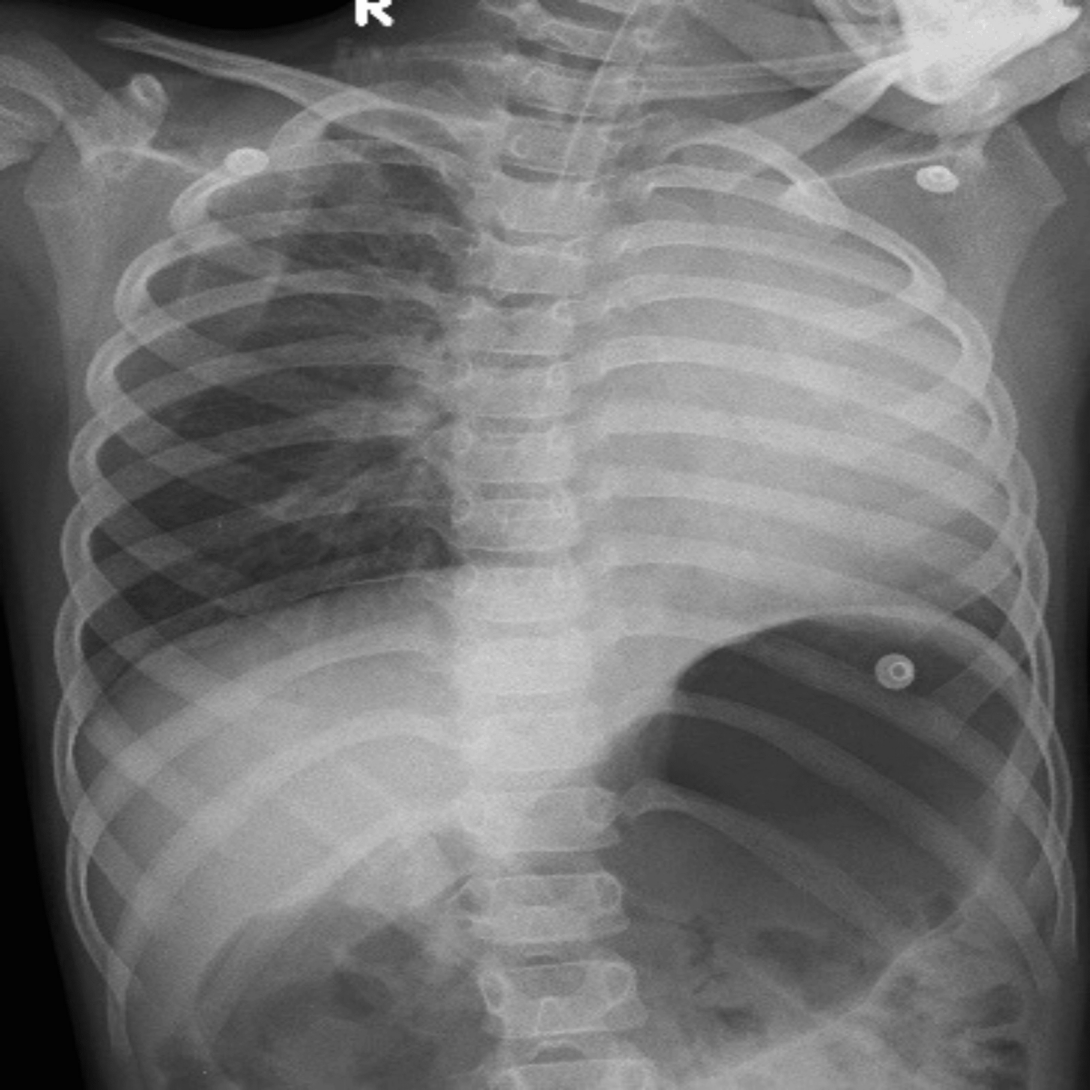
Chest Radiograph Demonstrating Left Lung Volume Loss Following Foreign Body Aspiration Chest radiograph obtained at presentation demonstrating left lung volume loss with compensatory hyperinflation of the contralateral lung following aspiration of a toy whistle component.

At presentation to our institution, the child was hemodynamically stable and maintained normal oxygen saturation on room air. He had a persistent cough but no fever, breathlessness, vomiting, wheeze, or other systemic symptoms. Respiratory examination revealed reduced air entry over the left hemithorax. A chest radiograph repeated at our institution demonstrated left lung volume loss with compensatory hyperinflation of the contralateral lung (Figure 1). CT images obtained at the referring hospital demonstrated an endobronchial foreign body within the left main bronchus with associated lingular atelectasis.

Considering the prior unsuccessful retrieval attempt, the need for distal airway visualization, and the anticipated technical difficulty of retrieval, a staged flexible bronchoscopic approach was selected with rigid bronchoscopy backup immediately available throughout the procedure. Flexible bronchoscopy was performed under general anesthesia through a laryngeal mask airway using a Fujifilm EB-710P slim bronchoscope (Fujifilm, Tokyo, Japan) with a 2.2 mm working channel. Ventilation was maintained through the laryngeal mask airway during the procedure. Continuous electrocardiographic, pulse oximetry, and capnography monitoring were maintained throughout both bronchoscopic sessions. No significant desaturation or hemodynamic instability occurred during either procedure.

**Figure 2:**
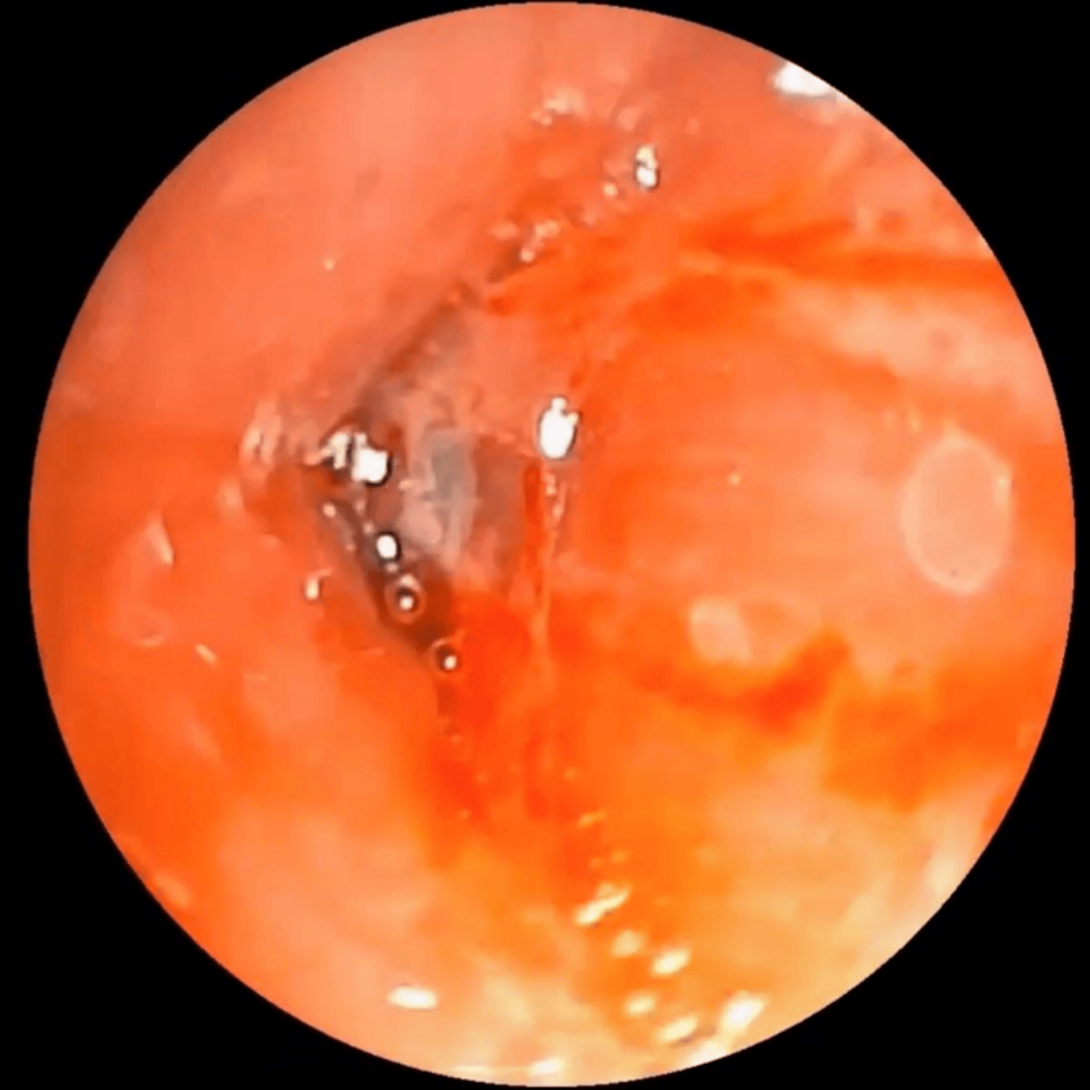
Bronchoscopic Visualization of the Retained Toy Whistle Component in the Left Main Bronchus Bronchoscopic image demonstrating the retained toy whistle component impacted in the left main bronchus with surrounding granulation tissue and airway inflammation during the initial procedure.

During the first bronchoscopy session, which lasted approximately 35 minutes, retained fragments of the toy whistle were visualized within the left main bronchus with surrounding granulation tissue and retained secretions, limiting visualization (Figure 2). Biopsy forceps were initially used; however, adequate purchase could not be achieved because of the smooth plastic surface. Alligator forceps and rat-tooth forceps were subsequently used, and several whistle fragments were successfully removed. However, complete clearance was not possible because a retained hollow cylindrical component remained impacted, while thick granulation tissue and retained secretions limited maneuverability and visualization.

Nebulized budesonide was administered for 24 hours between procedures based on institutional clinical practice and procedural judgment, with the aim of reducing airway inflammation and granulation tissue to improve visualization during repeat bronchoscopy. Repeat bronchoscopy was performed the following day. During the second bronchoscopy session, which lasted approximately 20 minutes, a reduction in granulation tissue permitted clearer visualization of the retained hollow cylindrical component within the left main bronchus. Further retrieval attempts using forceps were unsuccessful because the smooth hollow component repeatedly slipped during grasping attempts.

**Figure 3:**
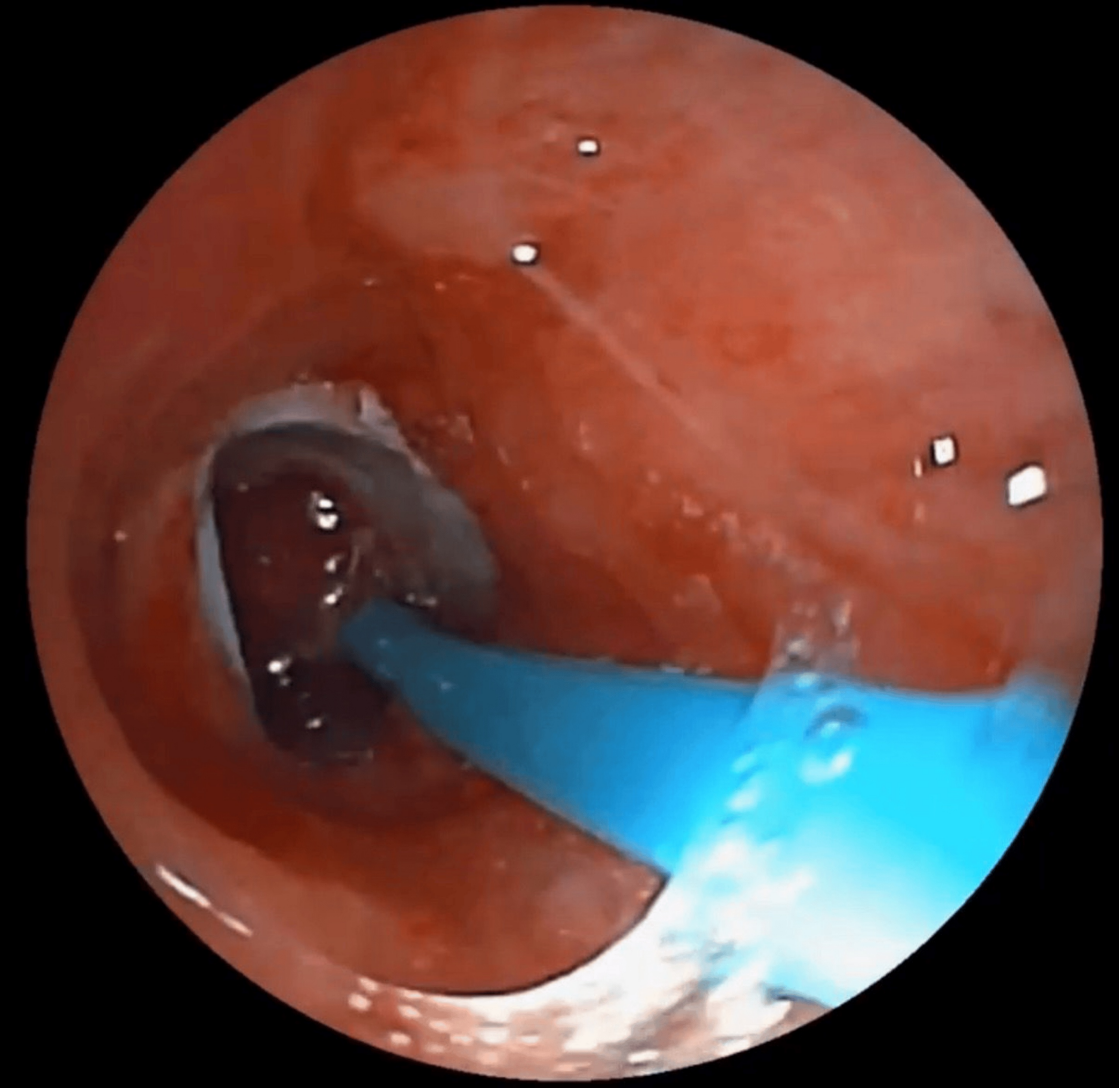
Fogarty Balloon Catheter-Assisted Retrieval of the Retained Hollow Toy Whistle Component Bronchoscopic image demonstrating a modified 4 Fr Fogarty balloon catheter passed through the retained hollow whistle component during repeat bronchoscopy. Balloon inflation with 0.5 mL saline enabled successful extraction after failure of air inflation.

A 4 Fr Fogarty embolectomy catheter was then advanced distal to the retained hollow component (Figure 3). Initial balloon inflation with air repeatedly failed to achieve adequate anchorage because of slippage within the smooth lumen. The balloon was subsequently inflated with 0.5 mL saline, which improved intraluminal apposition and enabled the successful extraction of the retained hollow cylindrical component with gentle traction.

**Figure 4:**
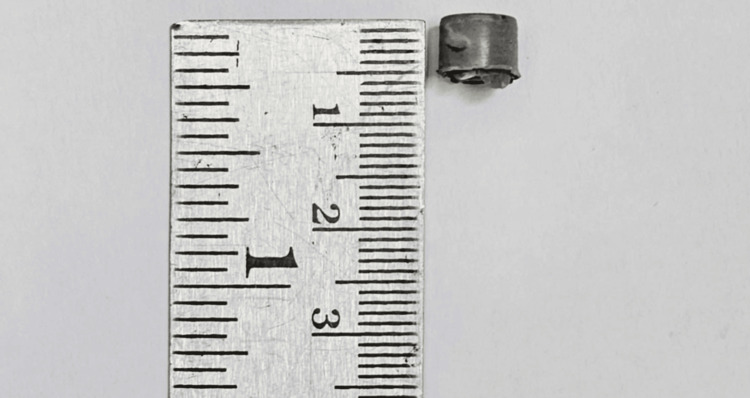
Retrieved Hollow Plastic Component of the Aspirated Toy Whistle Photograph of the retrieved hollow cylindrical plastic whistle component displayed alongside a measurement scale (approximately 0.6 cm in length).

**Figure 5:**
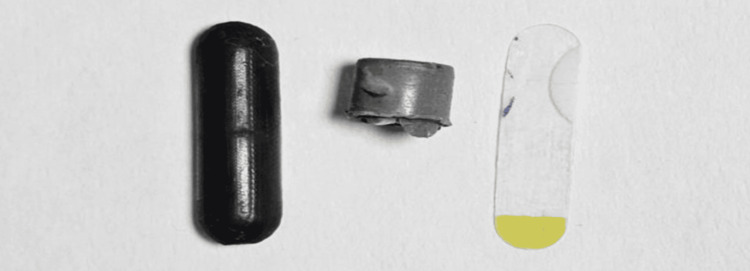
Retrieved Components of the Aspirated Toy Whistle Photograph showing the separated retrieved components of the aspirated toy whistle following successful bronchoscopic extraction. The image demonstrates the black cylindrical plastic body (left), hollow cylindrical plastic component (center), and internal whistle reed component (right).

The retrieved hollow cylindrical plastic component measured approximately 0.6 cm and is shown alongside a measurement scale (Figure 4). Complete retrieval of all aspirated toy whistle components, including the black cylindrical plastic body, hollow cylindrical plastic component, and internal whistle reed component, was subsequently confirmed (Figure 5).

**Figure 6:**
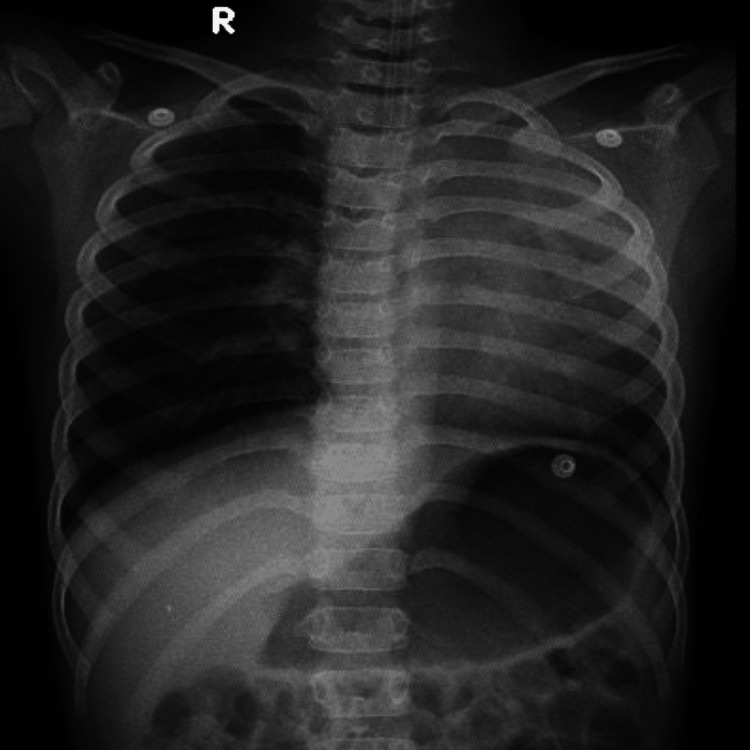
Post-procedure Chest Radiograph Demonstrating Re-expansion of the Left Lung Follow-up chest radiograph demonstrating interval improvement in left lung aeration and re-expansion following successful retrieval of the aspirated toy whistle components.

Post-procedure bronchoscopy demonstrated mild mucosal edema without significant bleeding or residual foreign body. Follow-up chest radiography demonstrated re-expansion of the left lung with improved aeration compared with the pre-procedure radiograph (Figure 6). The child remained clinically stable, tolerated the procedure without complications, and was discharged in satisfactory condition.

## Discussion

FBA remains a significant cause of respiratory morbidity in children, particularly when diagnosis is delayed or retrieval is technically challenging. Although organic foreign bodies are more commonly encountered, smooth plastic foreign bodies may pose unique bronchoscopic challenges because they are difficult to grasp securely and are prone to repeated slippage during extraction [1-3]. Delayed presentation may further complicate management because of secondary airway changes such as granulation tissue formation, retained secretions, recurrent infection, and atelectasis. In the present case, delayed presentation was associated with persistent cough, lingular atelectasis, retained secretions, and granulation tissue formation, all of which increased procedural complexity and limited visualization during bronchoscopy.

Rigid bronchoscopy remains the standard modality for pediatric airway foreign body retrieval because it provides superior airway control, effective ventilation, and access to a broad range of retrieval instruments [4,5]. However, flexible bronchoscopy has an increasingly recognized adjunctive role in selected clinical scenarios, particularly in experienced centers when distal airway visualization, technically difficult retrieval, staged procedures, or minimally invasive approaches are desirable [6,7]. In the present case, rigid bronchoscopy backup was immediately available throughout the procedure; however, a staged flexible bronchoscopic approach was selected because the child had already undergone an unsuccessful retrieval attempt at another institution, improved distal airway visualization was required, and stepwise extraction was considered technically feasible while maintaining immediate airway backup if escalation became necessary.

The retrieval process in this patient was technically demanding because the aspirated toy whistle fragmented into multiple components. During the first bronchoscopy session, which lasted approximately 35 minutes, several retained fragments were successfully removed using alligator and rat-tooth forceps after biopsy forceps failed to obtain adequate purchase on the smooth plastic surface. However, complete retrieval could not be achieved because a retained hollow cylindrical component remained impacted within the left main bronchus, while surrounding granulation tissue and retained secretions significantly limited visualization and maneuverability. A staged strategy was therefore adopted. Nebulized budesonide was administered for 24 hours between procedures based on institutional clinical practice and procedural judgment, with the aim of reducing airway inflammation and granulation tissue to improve visualization during repeat bronchoscopy. Although improved visualization was observed during the second procedure, this finding should be interpreted cautiously, given the single-case nature of this report and the inability to establish causality.

Fogarty balloon catheters are established adjunctive tools in airway foreign body retrieval, particularly for hollow or ring-shaped foreign bodies in which a “pass-through” technique may be used. Conventionally, the catheter is advanced distal to the foreign body and inflated with air to facilitate traction during withdrawal [8]. Previous reports have demonstrated the utility of Fogarty balloon-assisted retrieval in technically difficult airway foreign bodies, particularly when forceps alone fail to achieve adequate purchase [8]. Alternative strategies described for smooth or hollow foreign bodies include rigid bronchoscopy-assisted extraction, grasping forceps, basket retrieval devices, suction-assisted removal, and combined techniques depending on foreign body morphology and airway accessibility [4-8]. Nevertheless, smooth hollow plastic foreign bodies remain particularly challenging because repeated slippage may occur despite apparently adequate balloon positioning.

During the second bronchoscopy session, which lasted approximately 20 minutes, a reduction in granulation tissue permitted clearer visualization of the retained hollow component. Further attempts using forceps remained unsuccessful because the object repeatedly slipped during grasping. Initial Fogarty balloon inflation with air also repeatedly failed because of inadequate anchorage within the smooth lumen of the retained whistle component. Inflation with 0.5 mL saline was therefore used as a practical troubleshooting modification to improve intraluminal apposition and anchorage while avoiding escalation to more invasive retrieval strategies. This adjustment ultimately enabled the successful extraction of the retained component without procedural complications or hemodynamic instability.

We do not propose saline inflation of a Fogarty balloon catheter as a novel technique, as Fogarty-assisted retrieval and saline-filled balloons are familiar concepts in interventional practice. Rather, our experience highlights a practical troubleshooting modification that may be considered when conventional air inflation fails in selected hollow airway foreign bodies. This observation may be clinically useful for bronchoscopists encountering similar technical difficulties during pediatric airway foreign body retrieval; however, broader applicability remains uncertain.

A limitation of this report is its single-case nature, which limits generalizability. Furthermore, the observed improvement in visualization after nebulized budesonide administration cannot be definitively attributed to the intervention alone and should be interpreted cautiously. Additional reports or larger case series would be required to better define the reproducibility and clinical utility of saline-inflated Fogarty balloon assistance in technically difficult hollow airway foreign bodies.

This case further emphasizes the importance of individualized procedural planning, staged bronchoscopic management, and immediate availability of airway backup in technically difficult pediatric FBA. The successful outcome avoided escalation to more invasive intervention and resulted in complete lung re-expansion without significant complications.

## Conclusions

This case demonstrates that staged flexible bronchoscopy can be an effective approach for technically difficult pediatric airway foreign body retrieval in selected settings with appropriate expertise and rigid bronchoscopy backup. Smooth hollow plastic foreign bodies may present unique retrieval challenges because of inadequate forceps grip and repeated balloon slippage. In this case, saline inflation of a 4 Fr Fogarty balloon catheter provided improved intraluminal anchorage after failure of conventional air inflation, enabling the successful removal of a retained toy whistle component. Although not a novel technique, this practical troubleshooting modification may be considered in selected hollow airway foreign bodies when conventional retrieval methods are unsuccessful. Further experience is needed to better define its utility in similar technically challenging cases.
